# Biodegradation of Chloroquine by a Fungus from Amazonian Soil, *Penicillium guaibinense* CBMAI 2758

**DOI:** 10.3390/jof11080579

**Published:** 2025-08-04

**Authors:** Patrícia de Almeida Nóbrega, Samuel Q. Lopes, Lucas S. Sá, Ryan da Silva Ramos, Fabrício H. e Holanda, Inana F. de Araújo, André Luiz M. Porto, Willian G. Birolli, Irlon M. Ferreira

**Affiliations:** 1 Biocatalysis and Applied Organic Synthesis Laboratory, Federal University of Amapá, Macapá 68903-419, AP, Brazil; doutoradobiotecnologia21@gmail.com (P.d.A.N.); samuql12@gmail.com (S.Q.L.); lucassa720@gmail.com (L.S.S.); inanafauro24@gmail.com (I.F.d.A.); 2 Post-Graduate Program in Biotechnology and Biodiversity—BIONORTE Network, Federal University of Amapá, Macapá 68902-280, AP, Brazil; ryan.ramos@ueap.edu.br; 3 Federal Institute of Science and Technology Education of Amapá, Rua Nilo Perçanha, Bairro Cajari, Laranjal do Jari 68920-000, AP, Brazil; holanda_fabricio@yahoo.com.br; 4 Laboratory of Organic Chemistry and Biocatalysis, São Carlos Institute of Chemistry, University of São Paulo, Ed. Prof. Douglas Wagner Franco, Av. João Dagnone 1100, Santa Angelina, São Carlos 13563-120, SP, Brazil; almporto@iqsc.usp.br; 5 Molecular Oncology Research Center, Educational and Research Institute, Barretos Cancer Hospital, Av. Ébano 165-1, Dr. Paulo Prata, Barretos 14784-384, SP, Brazil; willianbirolli@gmail.com

**Keywords:** Amazonian fungi, biodegradation, pharmaceutical compounds, environmental pollution, COVID-19

## Abstract

Concern over the presence of pharmaceutical waste in the environment has prompted research into the management of emerging organic micropollutants (EOMs). In response, sustainable technologies have been applied as alternatives to reduce the effects of these contaminants. This study investigated the capacity of filamentous fungi isolated from iron mine soil in the Amazon region to biodegrade the drug chloroquine diphosphate. An initial screening assessed the growth of four fungal strains on solid media containing chloroquine diphosphate: *Trichoderma pseudoasperelloides* CBMAI 2752, *Penicillium rolfsii* CBMAI 2753, *Talaromyces verruculosus* CBMAI 2754, and *Penicillium* sp. cf. *guaibinense* CBMAI 2758. Among them, *Penicillium* sp. cf. *guaibinense* CBMAI 2758 was selected for further testing in liquid media. A Box–Behnken factorial design was applied with three variables, pH (5, 7, and 9), incubation time (5, 10, and 15 days), and chloroquine diphosphate concentration (50, 75, and 100 mg·L^−1^), totaling 15 experiments. The samples were analyzed by gas chromatography–mass spectrometry (GC-MS). The most effective conditions for chloroquine biodegradation were pH 7, 100 mg·L^−1^ concentration, and 10 days of incubation. Four metabolites were identified: one resulting from *N*-deethylation M1 (*N*4-(7-chloroquinolin-4-yl)-*N*1-ethylpentane-1,4-diamine), two from carbon–carbon bond cleavage M2 (7-chloro-*N*-ethylquinolin-4-amine) and M3 (*N*1,*N*1-diethylpentane-1,4-diamine), and one from aromatic deamination M4 (*N*1-ethylbutane-1,4-diamine) by enzymatic reactions. The toxicity analysis showed that the products obtained from the biodegradation of chloroquine were less toxic than the commercial formulation of this compound. These findings highlight the biotechnological potential of Amazonian fungi for drug biodegradation and decontamination.

## 1. Introduction

The COVID-19 pandemic, caused by the SARS-CoV-2 virus, triggered a global public health crisis that demanded rapid and innovative responses. In this context, repositioning of drugs, also known as repurposing, has gained prominence as a strategy for accelerating the development of treatments, given that the safety profiles for these drugs have already been established. As a result, repositioning became an essential tool in the search for effective therapies against COVID-19 [1].

**Figure 1:**
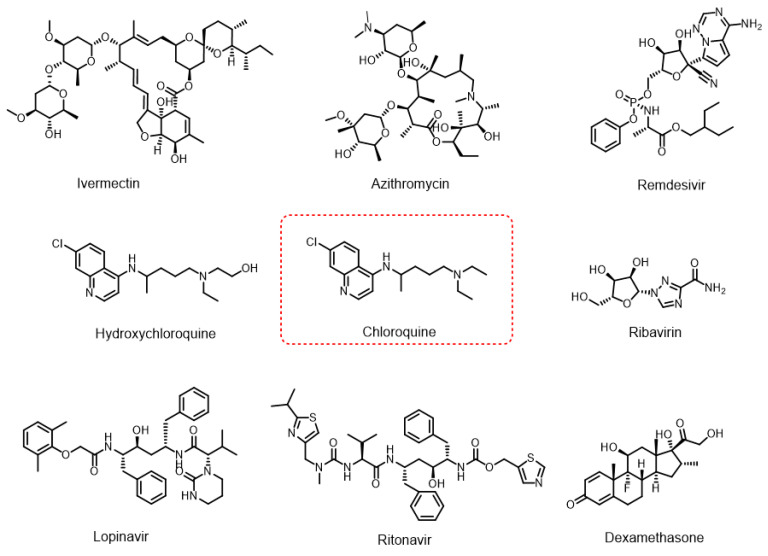
Chemical structures of selected drugs investigated as potential treatments for COVID-19 during the pandemic.

During the pandemic, numerous drugs used to treat other diseases were tested as potential therapies for COVID-19 [2], in some cases without robust scientific evidence. Among the most widely used were hydroxychloroquine [3], chloroquine [4], ivermectin [5], azithromycin [6], favipiravir [7], remdesivir [8], lopinavir–ritonavir, ribavirin, and dexamethasone [9] (Figure 1).

Increased consumption and incorrect disposal of these drugs during the COVID-19 pandemic generated worrying impacts on public health and the environment, with an increased risk of environmental contamination [10,11]. This risk mainly arises from the potential adverse effects that pharmaceutical waste can have on humans and on aquatic and terrestrial ecosystems [11,12,13]. Much of this waste enters the environment through domestic, industrial, and hospital effluents, as most drugs are not fully metabolized in the human body and are excreted in urine or feces, either unchanged or as active metabolites [14,15].

A study reported significant increases in the concentrations of drugs, including those used against COVID-19, in aquatic environments during the pandemic in comparison with pre-pandemic levels [16]. This highlights how the intensified use of pharmaceuticals during the health crisis exacerbated the issue of environmental pollution by these compounds as micropollutants. In particular, drugs such as chloroquine present a concern in wastewater treatment plants and natural water bodies, as they may not be fully removed during these processes and can enter the environment due to their moderate persistence [10]. Furthermore, certain pharmaceutical compounds can become highly toxic when improperly disposed, posing significant risks to human health and ecosystems, even at low concentrations [17,18].

The removal of pharmaceutical compounds from the environment using conventional chemical and physical treatments is often inefficient, unsustainable, or economically unfeasible. In recent years, a number of sustainable technologies have been developed to manage pharmaceutical organic micropollutant (OMP) waste. These methods include the use of oxidation–reduction techniques, membranes [19], adsorption [20], photodegradation [21], and biodegradation [22,23,24].

Although still limited in number, some studies have demonstrated the degradation of chloroquine by physical and chemical methods. For example, Midasi et al. [25] compared oxidation methods and observed that the electro–Fenton–BDD process was the most efficient, achieving complete elimination of chloroquine and 92% removal of total organic carbon (TOC). Dai et al. [10] evaluated the degradation of chloroquine phosphate (CQP) and found that the UV/H_2_O_2_ process outperformed the isolated use of UV or H_2_O_2_. Similarly, Han Qi et al. [26] tested ozonation for the degradation of low concentrations of chloroquine phosphate (0.1 mg/L), and under ideal conditions (pH 7, 30 °C, and 0.192 mg/L ozone), complete removal was achieved within 40 min.

Biodegradation is the process by which organic substances are broken down by microorganisms into simpler natural compounds (e.g., CO_2_, H_2_O, and NH_3_) that can be integrated into natural biogeochemical cycles. The biodegradation of pharmaceuticals by microorganisms such as fungi and bacteria has emerged as an effective, environmentally sustainable, and cost-efficient alternative for the removal of organic micropollutants [24,27,28].

Several studies have documented the use of microorganisms in the process of biodegradation and biotransformation of pharmaceuticals. For example, a recent study demonstrated the effectiveness of *Aspergillus* sp., isolated from activated sludge, in degrading chlortetracycline, achieving a biodegradation rate of 95% after 72 h under controlled conditions [29,30,31]. Likewise, a strain of the filamentous fungus *Cunninghamella echinulata* var. *elegans* (ATCC 8688a) grown in a medium containing hydroxychloroquine biotransformed this compound after 96 h of incubation, producing various metabolites. The main metabolite identified was 4-(1,2,3,4-tetrahydroquinoline-4-yl amino)pentan-1-ol [32].

Among the promising fungi in biotechnological processes, those found in the Amazon Rainforest, though still little studied, stand out for their potential [33]. The forest’s ecological richness is sustained by its microbial diversity, which plays vital roles in recycling organic matter and supporting both plant and animal life. In particular, metabolically active soil fungi produce valuable compounds that can be harnessed for processes such as biodegradation [34] and biotransformation [24].

In this context, and with the aim of advancing the knowledge of the enzymatic potential of fungi for the biodegradation of chloroquine, this study investigated the ability of filamentous fungi isolated from Brazilian Amazonian soil to degrade this pharmaceutical product.

## 2. Results and Discussion

### 2.1. Identification of Filamentous Fungi

The fungi used in the biodegradation process were previously isolated and identified [35]. Species identification was performed using macromorphological, micromorphological, and molecular analyses, resulting in the identification of *Trichoderma pseudoasperelloides* CBMAI 2752, *Penicillium rolfsii* CBMAI 2753, *Talaromyces verruculosus* CBMAI 2754, and *Penicillium* sp. cf. *guaibinense* CBMAI 2758. Figure S1 (Supplementary Materials) shows the fungal isolates grown on Sabouraud dextrose agar and 2% malt extract agar (pH 5) in Petri dishes, incubated at approximately 28 °C for 7 days. All fungi were identified at the Multidisciplinary Center for Chemical, Biological, and Agricultural Research (CPQBA) from Unicamp (https://site.cpqba.unicamp.br/).

The soil of the Amazon region stands out due to its remarkable microbiological diversity, hosting a wide variety of microorganisms with biotechnological potential [36]. Numerous studies have explored this rich microbial reservoir, focusing on the characterization and isolation of species, especially filamentous fungi, that play key roles in ecological processes and offer promising applications in biodegradation, biocontrol, and the production of industrially relevant metabolites.

Radial growth assays were conducted to preliminarily evaluate the effects on filamentous fungal strains isolated from iron mine soil. Chloroquine diphosphate at the concentrations of 25, 50, and 100 mg·L^−1^ was added to the strains *T. pseudoasperelloides* CBMAI 2752, *P. rolfsii* CBMAI 2753, *T. verruculosus* CBMAI 2754, and *P.* sp cf*. guaibinense* CBMAI 2758 on solid culture media (2% Sabouraud dextrose agar and 2% malt). Colony growth was measured every 24 h over a seven-day period. Plates of solid media without chloroquine diphosphate were used as fungal control (biotic). The experiments were carried out in triplicate [24].

**Figure 2:**
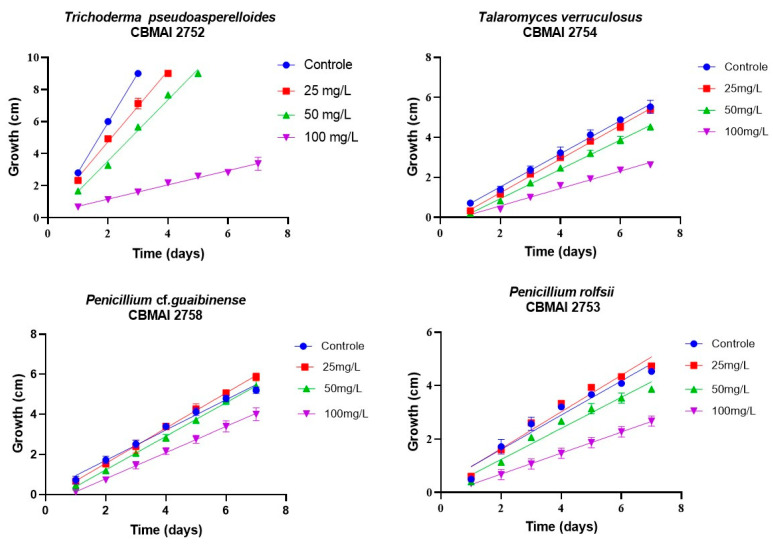
Radial growth of fungal strains isolated from iron mine soil cultured on solid media (Sabouraud dextrose agar and 2% malt, pH 5, at ± 28 °C for 7 days).

The results for mycelial growth, based on the average diameter (cm) in the Petri dish, are shown in Figure 2. Radial growth rates were determined by applying linear regression to the data for all tested species. Three strains (*T. verruculosus* CBMAI 2754, *P. rolfsii* CBMAI 2753, and *P.* sp cf. *guaibinense* CBMAI 2758) showed adaptive growth behavior in the presence of chloroquine diphosphate. Among these, *P.* sp cf*. guaibinense* CBMAI 2758 was particularly notable, displaying enhanced growth when cultivated in medium enriched with this substance at 25 mg·L^−1^ and 50 mg·L^−1^, as evidenced by larger colony diameters in comparison with the control group.

The radial growth velocity or radial growth rate (RGR) corresponds to the slope of the line obtained from linear regression of the colony radius over time. A greater slope indicates a higher RGR and, consequently, greater fungal growth potential [37]. Based on the RGR results, *Talaromyces verruculosus* CBMAI 2754, *Penicillium* cf. *guaibinense* CBMAI 2758, and *Penicillium rolfsii* CBMAI 2753 exhibited higher growth rates in medium containing 25 mg·L^−1^ chloroquine diphosphate compared to the control medium (absence of chloroquine diphosphate).

In contrast, *T. pseudoasperelloides* CBMAI 2752 showed an RGR of 2.22 cm/d for the medium enriched with chloroquine and 3.10 cm/d for the medium in the absence of chloroquine. Therefore, the presence of chloroquine in the medium led to a reduction in the radial growth rate at all tested concentrations. In contrast, *Penicillium* sp. cf. *guaibinense* CBMAI 2758 appeared capable of utilizing chloroquine as a carbon source to support its mycelial growth. These findings suggest that this microorganism holds significant potential as a biotechnological agent for the biodegradation of chloroquine. Detailed growth data are provided in Table S1 (Supplementary Material).

Pearson’s correlation analysis comparing the growth rates of the fungal strains with varying concentrations of chloroquine diphosphate yielded a correlation coefficient of −0.520. This moderate negative correlation indicates that as the concentration of chloroquine diphosphate increases, fungal growth tends to decrease, demonstrating the inhibitory effect of the drug on fungal development. In the experiments with *T. verruculosus* CBMAI 2754 and *P. rolfsii* CBMAI 2753, the diameter of the strains treated with 25 mg·L^−1^ did not differ significantly from those of the control. This suggests that chloroquine diphosphate does not interfere with the growth of these strains at this concentration.

However, it is important to emphasize that further studies are needed to assess the effects of longer exposure periods. *T. verruculosus* CBMAI 2754, *P. rolfsii* CBMAI 2753, and *T. pseudoasperelloides* CBMAI 2752 showed differences in colony coloration when grown in the presence of chloroquine diphosphate, as shown in Table 1. Notably, *T. pseudoasperelloides* CBMAI 2752 showed significant inhibition of growth with 100 mg·L^−1^ chloroquine diphosphate in comparison with the control experiment (Table 1). This inhibition may have been the result of a harmful effect caused by the drug itself or from metabolites produced during its biodegradation.

### 2.2. Evaluation of the Biodegradation of Chloroquine Diphosphate Through Experimental Design

**Figure 3:**
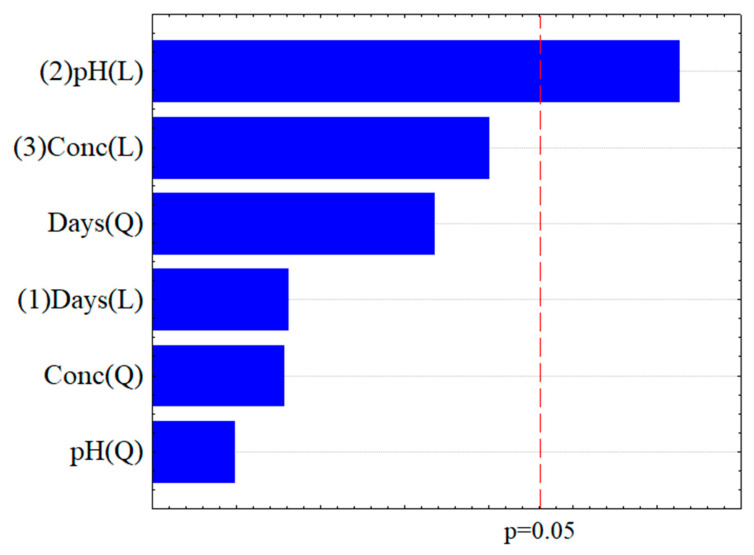
Pareto diagram of the linear (L) and quadratic (Q) effects and order of factors (1, time; 2, pH; 3, and concentration) on the biodegradation of chloroquine (%) by the fungal strain *Penicillium* cf. *guaibinense* CBMAI 2758.

The biodegradation process was evaluated based on the percentage reduction of chloroquine diphosphate, as determined by the experimental design using *P*. cf. *guaibinense* CBMAI 2758 as biodegradation agent. The variables’ values are summarized in Table 2. A Pareto diagram of standardized effects was generated for the tests with this microorganism (Figure 3) and, although the diagram presents all tested effects, only pH was statistically significant, as indicated by the reference significance line.

In the Pareto diagram, the length of each bar represents the magnitude of the estimated effect for each parameter, while the vertical line indicates the threshold for statistical significance at the 5% level. Within the experimental range tested, the variables that exceeded this threshold were considered statistically significant at a 95% confidence level (*p* < 0.05), as shown in Figure 3 [24].

According to Figure 3, pH was the only variable with a statistically significant effect on the biodegradation of chloroquine diphosphate by *P*. cf *guaibinense* CBMAI 2758. Notably, pH emerged as an important factor, with neutral conditions (pH 7) yielding the highest degradation percentages.

Fungi have different nutritional and environmental requirements, which include their preference for an optimal pH range for growth. This pH preference can vary among species and depend on the specific environmental conditions in which the fungi develop. However, most fungi thrive in slightly acidic environments, typically around pH 5 to 6. This range is favorable to many filamentous fungi and yeasts, enabling adequate growth and development [38,39].

According to Rigo et al. [40], variables such as pH, temperature, humidity, and nutrient availability play a decisive role in biotechnological processes, affecting microbial growth and reaction efficiency. The author also emphasizes that factors such as medium composition, incubation time, and substrate concentration are key determinants for optimizing these processes.

**Figure 4:**
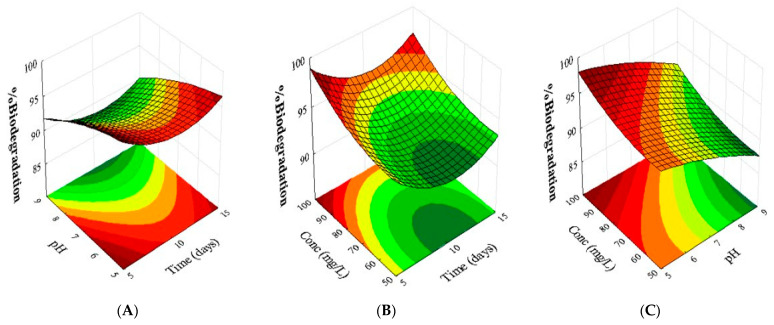
Response surface plots for the biodegradation of chloroquine diphosphate by the fungus *Penicillium* sp. cf. *guaibinense* CBMAI 2758 as a function of (**A**) the reaction time (X1) and pH of the culture medium (X2); (**B**) reaction time (X1) and chloroquine diphosphate concentration (X3); and (**C**) pH of the culture medium (X2) and chloroquine diphosphate concentration (X3).

Figure 4 presents the projection of three response surface graphs used to optimize the biodegradation conditions. The quadratic model demonstrated a significant correlation between the response and the factors time, pH, and concentration. These graphs allow an analysis of the interactions between pairs of variables, providing a detailed evaluation of their combined effects on the biodegradation efficiency [41].

The biodegradation of chloroquine diphosphate by the strain *P.* sp. cf. *guaibinense* CBMAI 2758 was influenced by the following variables: time and pH of the reaction medium (Figure 4A), the interaction between time and concentration (Figure 4B), and the interaction between pH and concentration (Figure 4C). Among these variables, the analysis of the incubation time (evaluated at 5, 10, and 15 days) showed that 10 days had the most significant positive effect on biodegradation. Thus, extending the incubation period beyond 10 days did not lead to further meaningful increases in the degradation rate, indicating that longer durations offered no added benefit. Furthermore, an analysis of the interaction between time and pH revealed that decreasing the pH below 7 significantly reduced the biodegradation rate, whereas increasing it above 7 did not increase the biodegradation rate significantly. Therefore, the response surface graph confirmed that the optimal conditions, considering the combined effects of these variables, were 10 days of incubation at pH 7.

A comparison of the variables concentration versus time showed that increasing the concentration of chloroquine diphosphate led to higher biodegradation rates, reaching up to 100%, which indicates that this was the most significant variable alongside time. An increase or decrease in time above or below 10 days did not greatly influence the biodegradation rate. Thus, 10 days remained the optimal time, as observed in graph A, which shows the interaction between pH and time.

In the analysis of pH versus concentration (mg·L^−1^), it was observed again that concentration was the variable that most influenced the biodegradation rate, reaching the optimal performance at 100 mg·L^−1^. Deviations from pH 7, either higher or lower, led to a reduction in biodegradation efficiency, confirming that pH 7 was the optimal condition for the process studied.

It is important to highlight that the solubility of chloroquine, especially in water at an adequate temperature and pH, can facilitate its availability for microbial activity, enhancing the biodegradation process. According to Daneshfar and Vafafard [42], the solubility of chloroquine diphosphate increases with temperature, reaching its maximum between 298.2 and 333.2 K. Thus, environments where chloroquine is more soluble are more favorable for efficient biodegradation, reinforcing the importance of optimized conditions for the treatment of this compound.

This study demonstrated that the chloroquine diphosphate biodegradation level achieved in most experiments at pH 7 was higher than 90%, as shown in Table 3. Several studies have reported on the use of microorganisms for the biodegradation of micropollutants, offering several advantages in comparison with other physical and chemical methods [43]. The remarkable effectiveness of fungi in bioremediation is largely due to the production of a broad spectrum of intracellular enzymes, with an emphasis on the cytochrome P450 complex. This superfamily of monooxygenases, which uses a heme group as a cofactor, plays an essential role in the degradation of recalcitrant compounds. Through reactions such as deamination, dealkylation, dehalogenation, and hydroxylation, these enzymes promote structural modifications in pollutants, making them more susceptible to degradation and eventual mineralization [44,45].

In addition to intracellular enzymes, fungi also produce extracellular enzymes that contribute to the breakdown of complex organic molecules. Laccases and peroxidases, in particular, have been associated with the degradation of various xenobiotics [44,46]. However, specific evidence of their activity against chloroquine is not yet available in the literature. Nonetheless, the enzymatic diversity of fungi remains a key factor in their potential application for environmental bioremediation [45].

This metabolic versatility gives fungi a distinct advantage over other organisms in the remediation of contaminated environments, underscoring their biotechnological potential for use in sustainable decontamination processes. Furthermore, the ability of fungi to act under extreme pH and temperature conditions expands their applicability for bioremediation compared with other microorganisms such as bacteria, whose enzyme systems are often more restricted [43,45].

A study conducted to investigate the efficacy of the strain *Trichoderma pubescens* DAOM 166162 for treating amoxicillin-containing wastewater showed that this strain removed more than 98% of the antibiotic within 24 h, with a biodegradation efficiency of 70%. Degradation occurs through hydroxyl groups in extracellular polymeric substances (EPS), which help to disrupt the structure of amoxicillin in alkaline environments, thus highlighting the potential of *Trichoderma* for the bioremediation of pharmaceutical wastewater [47].

A study by Li et al. [48] revealed that the fungal strains *Trichoderma harzianum*-LJ245, *Penicillium oxilacum*-LJ302, and *Penicillium citrinum*-LJ318 were effective at the biodegradation of chlortetracycline (CTC) in crude residue. The degradation rate reached 96% with LJ245, 99% with LJ302, and 98% with LJ318 over a 40-day period, in addition to increasing the pH of CTC crude residue from 2.30 to 8.32. These findings emphasize the potential of these strains as effective treatments for pharmaceutical waste.

Another study investigated the biodegradation of a mixture of nonsteroidal anti-inflammatory drugs (NSAIDs), including celecoxib, diclofenac, and ibuprofen, using the fungal consortium *Ganoderma applanatum* and *Laetiporus sulphureus*. The results showed that this consortium achieved a degradation efficiency exceeding 99%. Enzymatic analysis revealed a significant increase in ligninolytic enzyme production during the degradation process, indicating the importance of these enzymes for the effective removal of NSAIDs from the environment [49].

### 2.3. Identification of Biodegradation Metabolites by GC-MS

**Figure 5:**
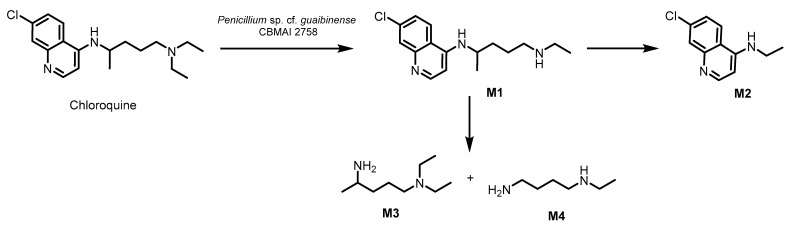
Proposal for the biodegradation of chloroquine by *Penicillium* sp. cf. *guaibinense* CBMAI 2758.

In the process of chloroquine diphosphate biodegradation by filamentous fungus from Amazonian soil, four metabolites were qualitatively identified. After 10 days of biodegradation by *Penicillium* sp. cf. *guaibinense* CBMAI 2758, the following metabolites were identified by GC-MS (mass spectra are presented in Table S2, Supplementary Material): M1, *N*4-(7-chloroquinolin-4-yl)-*N*1-ethylpentane-1,4-diamine, is a product of the action of *N*-deethylation with a molecular ion of *m*/*z* 290 generated from the loss of the *m*/*z* 29 ion (-CH_2_-CH_3_). The *N*-deethylation reaction is recognized as a detoxification pathway for environmental pollutants and the drug quetiapine [50]; M2, 7-chloro-*N*-ethylquinolin-4-amine, possibly results a carbon–carbon bond cleavage, producing a metabolite with *m*/*z* 205; whereas M3, *N*1,*N*1-diethylpentane-1,4-diamine, and M4, *N*1-ethylbutane-1,4-diamine, result from aromatic deamination (Figure 5).

### 2.4. Toxicological Analysis

Although chloroquine is widely employed for treating malaria and autoimmune diseases, it is associated with significant toxic effects on both human and rodent models, especially with prolonged exposure or at high doses. In humans, the main adverse effects include retinal toxicity, characterized by degeneration of the retinal pigment epithelium, which can lead to irreversible visual loss [51]. In addition, cardiac disorders have been reported, such as prolongation of the QT interval that can evolve into fatal arrhythmias [52]. In murine and rat models, studies have shown that hepatotoxicity, nephrotoxicity, and myocardial damage are associated with the accumulation of the drug in target tissues, as well as the induction of oxidative stress and cell apoptosis [53,54]. These findings emphasize the need for close monitoring during the clinical use of chloroquine and the importance of detailed preclinical evaluations using animal models.

The toxicological evaluation of the products derived from the biodegradation of chloroquine revealed a safety profile significantly superior to that of the parent drug. The results indicated a low risk of toxicity in relation to the carcinogenic potential, with TD_50_ values (median tumorigenic dose) ≥ 0.798 mg.kg^−1^ for rat models and ≥16.00 mg.kg^−1^ for mice. In addition, the maximum tolerated doses (MTD) in rats for the biodegradation products were higher than that of chloroquine, for which the value recorded was 0.016 g.kg^−1^, as shown in Table 4. These data suggest that the metabolites formed during the degradation process are less toxic, thus helping to reduce risks to human health and minimize the environmental impacts associated with the disposal of this pharmaceutical.

The *Penicillium* genus has been identified as a prolific producer of extracellular oxidative and hydrolytic enzymes, such as laccases, manganese peroxidases, lipases, proteases, esterases, and cellulases, that exhibit significant potential for the biodegradation of persistent organic compounds via molecular docking simulations [55,56]. For example, *Penicillium simplicissimum* secretes laccase (~66 kDa) and manganese peroxidase (~60 kDa), demonstrating effective activity in the degradation of polyethylene, as evidenced in spectrophotometric and FTIR/NMR studies. Tests with *Penicillium chrysogenum* demonstrated the production of laccase, lipase, and esterase under halophilic conditions, facilitating the decolorization of oily effluents [57].

Furthermore, molecular docking studies confirmed the interaction of fungal laccase and peroxidase with mycotoxins using structures with PDB IDs 1HFU and 1MNP, suggesting their potential in the degradation of aromatic-structured drugs [58,59,60]. Application of these enzymes, especially laccases and peroxidases, in chloroquine docking allows interactions in the active site to be simulated, identifying key residues (His, Asp, Cu, Ser, and Tyr) and anticipating possible mechanisms of enzymatic manipulation [55]. This approach supports the development of bioremediation strategies for pharmaceutical waste, as in silico evidence helps optimize the design of sustainable processes for environmental applications.

The results from the validation of the molecular docking simulation protocols were considered satisfactory, since the relative overlap (pose + orientation + torsion) of the crystallographic ligand (experimental control model) and the docking ligand (docking pose theoretical model) were considered similar. Recovering the pose of the complexes in Figure S2 (Supplementary Material), enabled validation of the docking protocols through the calculation of the root mean square deviation (RMSD). The average distances between atoms in the control molecules were 1.365, 1.433, and 0.642 Å, respectively.

**Figure 6:**
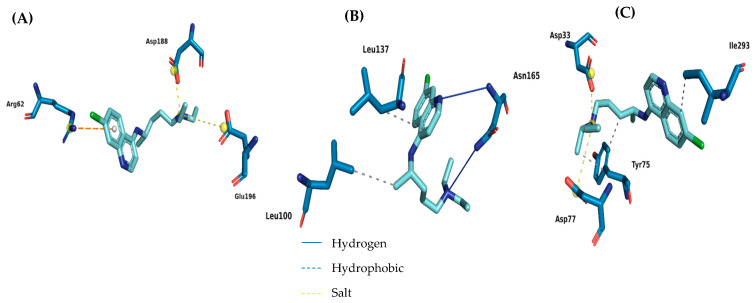
Map of the interactions of chloroquine with the receptors phytase (**A**), protease (**B**) and penicillopepsin-1 (**C**).

Extracellular phytases are generally absent in plants and animals, but are commonly isolated from bacteria and fungi. Protein overlap similarity analysis of 3-phytase from *Aspergillus niger* with *Penicillium* sp. indicated 63.68% shared identity in expressing the enzyme, and the three-dimensional structures of the proteins are virtually identical. According to Oakley [61], the 1′, 3′, 4′, 5′, and 6′-sulphate groups of the IHS bound to PhyA are in the equatorial conformation, while the 2′-sulphate group is in the axial position. This sulfate group also has the lowest β-factors among all six groups and is the most deeply inserted into the active site cavity. In addition, Arg58, Arg62, Arg142, and Asp339 interact with this group through hydrogen bonds and salt bridges. The molecular docking analysis of the chloroquine interaction showed a binding affinity of −7097 kcal/mol (Table 5), and similarity with the Arg62 amino acid residue (salt bridge type, Figure 6) located in the β-sheet.

The aspartic protease secreted by fungi of the *Penicillium* genus, notably *penicillopepsin*, has wide industrial and biotechnological use due to its high thermal stability, catalytic efficiency at acidic pH, and specificity for peptide bonds between hydrophobic residues [57]. In the environmental context, this enzyme contributes significantly to the biodegradation of nitrogen-containing organic residues, such as those present in industrial effluents, being considered an important tool for bioremediation processes and reuse of protein biomass. Its three-dimensional structure was one of the first to be elucidated among aspartic proteases, and it has become a model for mechanistic and enzyme engineering studies. In the study by Koszelak et al. [62], an ordered PMSF was found in the groove formed by the segments Leu137-Gly 138-Gly 139 and Gly 164-Asn 165-Glu 166 and had Ser173 and Asn198 below it. The molecular docking analysis in the present study showed that chloroquine interacted with the active site of the protease with a binding affinity of −7475 kcal/mol (Figure 6 and Table 5), with similarity to the Leu100 and Asn165 residues.

In penicillopepsin-1, the two catalytic aspartate residues (Asp33 and Asp213) reside in two similarly folded loops at the base of the hydrophobic substrate-binding groove and in front of the central ‘sheet’. These active enzyme structures contain catalytic water between the aspartates that deprotonate to initiate acid–base-catalyzed hydrolysis. The tip of the flap makes two hydrogen bonds in the main chain via the nitrogen atoms (NH) of the main structure of Gly76 and Asp 77 with PPi3, further anchoring the inhibitor to the active site. The molecular docking analysis in the present study showed interactions with similar amino acid residues in chloroquine with Asp33, Asp77 and Tyr 75 (Figure 5). The binding affinity value for the penicillopepsin-1 site with chloroquine was −7923 kcal/mol (Table 5).

## 3. Materials and Methods

### 3.1. Reagents and Solvents

Malt extract was purchased from Kasvi (São Paulo, Brazil), Sabouraud dextrose agar from Himedia (São Paulo, Brazil), and chloroquine diphosphate from Fundação Oswaldo Cruz (Rio de Janeiro, Brazil) (batch 19090737). Chloroquine of analytical grade (99%, CAS no. 50-63-5) was from Sigma-Aldrich (São Paulo, Brazil). Ethyl acetate (99.5%), dimethyl sulfoxide (97%), and methanol (99.8%) of HPLC grade were purchased from Sigma-Aldrich (São Paulo, Brazil), along with alpha-naphthol (99%) and diethylamine (99%). Na_2_SO_4_ (99%) was purchased from Reagen (São Paulo, Brazil).

### 3.2. Isolation and Identification of Filamentous Fungi

Four filamentous fungi (*Trichoderma pseudoasperelloides* CBMAI 2752, *Penicillium rolfsii* CBMAI 2753, *Talaromyces verruculosus* CBMAI 2754, and *Penicillium* cf. *guaibinense* CBMAI 2758) were isolated from soil on land owned by the company UNAMGEN Mining and Metallurgy, located in the municipality of Mazagão (state of Amapá, Brazil), as described in a previous study [35]. These strains were identified under a microscope in accordance with previously described methodology [41] and were used for the biodegradation of chloroquine diphosphate.

Molecular approaches were also applied to confirm these identifications at CPQBA-Unicamp. Genomic DNA from cultures was purified using the phenol DNA extraction protocol. Amplification of gene markers was performed using PCR methodology with the extracted genomic DNA as template. The primers (synthetic oligonucleotides) used for the PCR reaction were as follows: 728f and TEFIr targeting the TEF (translation elongation factor) region for CBMAI 2752; and Bt2a and Bt2b targeting the beta-tubulin region for isolates CBMAI 2753, CBMAI 2754, CBMAI 2756 and CBMAI 2758. The amplification product was column-purified (GFX PCR DNA and gel band purification kit, GE Healthcare, São Paulo, Brazil) and was directly subjected to sequencing using the ABI 3500XL series automated sequencer (Applied Biosystems, São Paulo, Brazil).

To perform the genetic distance analysis, the partial gene sequences obtained through the primers were assembled into a consensus (single consensus sequence combining the different fragments obtained), and then compared with the sequences of organisms from the GenBank (http://www.ncbi.nlm.nih.gov/) and CBS (https://wi.knaw.nl, accessed on 20 May 2022) databases. Sequences of microorganisms related to the unknown sample were subsequently selected for construction of a dendrogram. The DNA sequences were aligned using the CLUSTAL X software within BioEdit 7.2.6 and the genetic distance analyses were performed using the MEGA software, version 6.0. A distance matrix was calculated using the model and the dendrogram was constructed from the genetic distances using the neighbor-joining method, with bootstrap values calculated from 1000 resamples, in the MEGA 6.0 package.

### 3.3. Radial Growth of Strains in the Presence of Chloroquine Diphosphate (Solid)

Each strain was cultivated in solid culture media (2% Sabouraud dextrose agar and 2% malt) in pH 5 adjusted with HCl (10%) or KOH (10%) solutions. The solutions, flasks, and all material were sterilized in an autoclave (Phoenix, model AV-75, São Paulo, Brazil) at 121 °C for 20 min. Then, chloroquine diphosphate solutions at different concentrations (25, 50, and 100 mg·L^−1^) were solubilized in 600 µL of DMSO. The drug was transferred to an Erlenmeyer flask with the culture medium at 45 ± 5 °C to avoid thermal degradation [63]. The plates were inoculated at a central point with a nickel–chromium inoculation needle. The plates were then incubated at 28 °C (B.O.D. LUCADEMA^®^ model LUCA 161/03, Grupo Lucadema, São Paulo, Brazil). Radial growth was monitored every 24 h for 7 days using calipers to measure the diameter in perpendicular positions. Solid-medium plates without chloroquine diphosphate were used as fungal (biotic) controls. The experiments were performed in triplicate.

### 3.4. Experimental Design and Statistical Model

A three-level, three-variable Box–Behnken factorial design was applied to determine the optimal conditions for the biodegradation of chloroquine diphosphate using the filamentous fungus *Penicillium* sp. cf. *guaibinense* CBMAI 2758. The variables were reaction time (days), pH, and chloroquine diphosphate concentration (mg·L^−1^). The independent variables were designated as X1, X2, and X3, and their values are shown in Table 2.

The biodegradation rate (BD%) was employed as the response variable for determining the ideal conditions. STATISTICA^®^ software (version 10, Statesoft Inc., Tulsa, OK, USA, trial version, 2011) was used for experimental design and data analysis. Analysis of variance (ANOVA) was used to evaluate the effects of the independent variables and the significance of their interactions. A Pareto chart was used to assess the significance of the effects of the tested variables on the biodegradation rate.

### 3.5. Biodegradation Reaction of Chloroquine Diphosphate by the Fungus in Liquid Medium

In the screening on solid medium, *Penicillium* cf. *guaibinense* CBMAI 2758 exhibited superior growth, and was selected for quantitative biodegradation experiments in liquid medium, in accordance with the aforementioned design. Erlenmeyer flasks (125 mL) containing 50 mL of liquid medium (2% malt) at different pH values (5, 7, and 9), adjusted using a 10% HCl or KOH solution, were sterilized in an autoclave (20 min at 121 °C and 1 atm) and used for fungal cultivation. Inoculations were performed with six circular fragments (0.5 cm in diameter) taken from the solid culture. These cultures were then incubated in an orbital shaker (LUCADEMA^®^, model LUCA-222) for 5 days (28 °C, 130 rpm). The experiments were conducted as previously described, with slight modifications [27].

Then, solutions of chloroquine diphosphate with different final concentrations (50, 75 and 100 mg·L^−1^) were added, and the reactions were incubated in an orbital shaker for 5, 10, and 15 days at 28 °C and 130 rpm. All the biodegradation experiments were performed in triplicate. An abiotic control was performed with the medium containing chloroquine diphosphate at 50 mg·L^−1^ with pH 5, 7, and 9, without the presence of the fungus.

### 3.6. Extraction of Chloroquine Diphosphate and Its Metabolites

To extract chloroquine diphosphate, 0.025 mg of alpha-naphthol (internal standard) dissolved in 400 µL of ethanol and 50 mL of ethyl acetate was added to each experiment, resulting in a solution of ethyl acetate and culture medium (1:1), which was kept under vigorous magnetic stirring for 30 min. The fungal cells were then filtered. Subsequently, the mixture was extracted by liquid–liquid partitioning in three steps with ethyl acetate (3 × 30 mL). Soon afterwards, anhydrous sodium sulfate (Na_2_SO_4_) was added, and the solution was filtered again. The excess solvent was removed under reduced pressure using a rotary evaporator (Quimis Q344B, Diadema, Brazil), followed by drying in an oven for 40 h at 40 °C. The samples were then lyophilized [24].

### 3.7. Method Validation

The method was validated in accordance with the Collegiate Board Resolution no. 166 (RDC, 2017) of the Brazilian Health Surveillance Agency (ANVISA). Selectivity, sensitivity, linearity, precision, detection, and quantification limit were assessed in the validation of the chromatographic method. In the analysis of method selectivity, it was confirmed that the substances or metabolites present in the samples did not co-elute with chloroquine diphosphate in the GC-MS analysis. For method validation, an abiotic control experiment was performed with chloroquine in 2% malt culture medium.

The reactions were prepared in a sterile environment and extracted as described in Section 3.6. Extraction of Chloroquine Diphosphate and Its Metabolites. Lastly, the solutions were prepared for chromatographic analysis in a 2% diethylamine methanolic solution (GC-MS) [24]. Linearity was verified through a calibration curve composed of five chloroquine diphosphate solutions (50, 350, 650, 950, and 1250 mg·L^−1^). To determine the chloroquine diphosphate concentration, an internal standard method was performed, employing the following equation: c = ax + B, where c corresponds to the analyte concentration in mg·L^−1^, x refers to the analyte chloroquine area over the internal standard alpha-naphtol area, a is the angular coefficient, and B is the linear coefficient.

Accuracy was defined as the degree of agreement between the results of the employed method and the value accepted as true (RDC, 2017). Precision was expressed as the relative standard deviation (RSD), also known as coefficient of variation (CV), which was calculated according to the equation below:

CV = [SD/DMC] × 100

where SD refers to the standard deviation and DMC is the average concentration determined.

The relative precision was calculated using the accuracy percentage equation as follows:

Precision% = [Mean detected concentration/Nominal concentration]

The detection limit (LD) and quantification limit (LQ) were determined based on the obtained analytical curve, which took into consideration the confidence intervals of the linear regression.

### 3.8. Detection of Chloroquine Diphosphate and Metabolites Through Gas Chromatography–Mass Spectrometry (GC-MS) Analysis

The extracts obtained from the biodegradation process were analyzed in a Shimadzu/GC-2010 gas chromatograph (Kyoto, Japan) equipped with a Shimadzu/AOC-5000 autoinjector and coupled to Shimadzu MS-2010 plus mass spectrometer (GC-MS) in SCAN mode at 70 eV. The furnace was equipped with a DB-5 fused silica column (J&W Scientific, 30 mm × 0.25 mm × 0.25 μm, Levittown, PA, USA) and the carrier gas was helium (He) at 65 kPa. The analyses were performed using a flow rate of 3 mL/min and a split of 5 with the injector at 250 °C and the detector at 270 °C. The initial oven temperature was 90 °C for 2 min, and then increased to 280 °C at a heating rate of 6 °C/min. This temperature was maintained for 26 min, resulting in a total analysis time of 60 min [64]. The scan mode was *m*/*z* 40–500, and metabolite identification was performed using the Wiley 8, NIST 5, NIST 21, and NIST 107 spectral libraries [4].

### 3.9. Toxicity Predictions

Toxicity prediction tests were carried out using Discovery Studio 2015 software (Accelrys, Inc., San Diego, CA, USA) via the Toxicity Prediction by Komputer Assisted Technology (TOPKAT) function (BIOVIA, San Diego, CA, USA, 2024). The toxicological properties analyzed were the following: carcinogenicity in rodents (female mice and female rats), Ames mutagenicity, skin irritation, and skin sensitization. Toxicity risk prediction calculations were carried out via TOPKAT and measured the following parameters: oral LD_50_ (g/kg body weight), *Daphnia magna* EC_50_ (mg/L), lowest dose at which a toxic or adverse effect is observed (LOAEL, g/kg body weight), and *Pimephales promelas* LC_50_ (g/L) [65]. In addition, the carcinogenic potential was also predicted using the parameters TD_50_ (mg/kg body weight/mouse-day) and maximum tolerated dose in rats (MRTD) [66].

### 3.10. Molecular Docking Simulations

Crystal structures were downloaded from the Protein Data Bank (PDB) with the ID codes 3K4Q (*Aspergillus niger*-phytase), 5Z6O (*Penicillium cyclopium*-protease), and 1BXO (penicillopepsin-1) with resolutions of 2.20, 1.70, and 0.50 Å, respectively [61,62,67]. The crystal structures were elucidated using the X-ray diffraction method and the controls myo-inositol (IHS), phenyl metasulfonic acid (PMS), and phosphonate inhibitor (PP7) were used to validate the molecular docking protocols.

Molecular docking simulations were carried out using the DockThor server (https://dockthor.lncc.br/v2/, accessed on 22 April 2025) [68]. The parameters of the algorithm used were set as follows: (1) 12 runs of docking conformations, (2) 500,000 evaluations per docking run, and (3) a population of 750 individuals. The quality of the protein–ligand docking score was evaluated based on the root mean square deviation (RMSD) between the best score of the docking pose and the experimental binding mode of the crystallographic ligand.

## 4. Conclusions

Among the four fungal strains investigated, *Talaromyces verruculosus* CBMAI 2754, *Penicillium* sp. cf. *guaibinense* CBMAI 2758, and *Penicillium rolfsii* CBMAI 2753 demonstrated better adaptation to the presence of chloroquine diphosphate. At a concentration of 25 mg·L^−1^, these strains exhibited higher radial growth rates (RGRs) than those observed in the control medium without this compound. *Penicillium* sp. cf. *guaibinense* CBMAI 2758 stood out by demonstrating superior growth at both 25 and 50 mg·L^−1^ chloroquine diphosphate compared to the control, highlighting its potential for application in biodegradation processes. Based on these results, this strain was selected for the optimization experiments involving time, pH, and xenobiotic concentration using a Box–Behnken factorial design. Analyses of the Pareto diagram and response surface graphs indicated that the pH and chloroquine diphosphate concentration were the most significant variables influencing the biodegradation process. The optimal conditions for maximizing biodegradation efficiency were identified as pH 7, an incubation time of 10 days, and concentration of 100 mg·L^−1^.

The GC-MS analyses identified four main metabolites of chloroquine diphosphate formed through *N*-deethylation M1 (*N*4-(7-chloroquinolin-4-yl)-*N*1-ethylpentane-1,4-diamine), carbon-carbon bond cleavage M2 (7-chloro-*N*-ethylquinolin-4-amine) and M3 (*N*1,*N*1-diethylpentane-1,4-diamine), and aromatic deamination M4 (*N*1-ethylbutane-1,4-diamine) by enzymatic reactions, thus confirming the biodegradation process.

The toxicological assessment revealed that the biodegradation of chloroquine produces compounds with a significantly improved safety profile than the parent drug. These results indicate lower toxicity, underscoring the potential of the biodegradation process as an effective and environmentally safer approach for treatment and disposal of chloroquine.

The filamentous fungi evaluated in this study demonstrated effective biodegradation of the drug chloroquine diphosphate, highlighting the potential of Amazonian biodiversity as a valuable source of microorganisms for developing sustainable technologies to treat organic micropollutants.

## Figures and Tables

**Table 1:** Radial growth of *T. pseudoasperelloides* CBMAI 2752, *P. rolfsii* CBMAI 2753, *T. verruculosus* CBMAI 2754, and *P.* sp cf. *guaibinense* CBMAI 2758 in the presence of chloroquine diphosphate.

Fungus	Growth Medium	Time (Days)		Growth Speed (cm/d)
		3	6	
*Trichoderma pseudoasperelloides* CBMAI 2752	Control	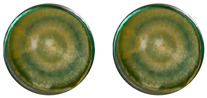		3.10
	Chloroquine 25 mg·L^−1^	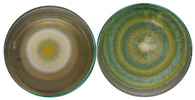		2.22
	Chloroquine 50 mg·L^−1^	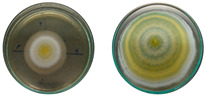		1.9
	Chloroquine 100 mg·L^−1^	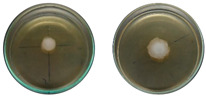		0.44
*Penicillium rolfsii* CBMAI 2753	Control	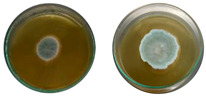		0.82
	Chloroquine 25 mg·L^−1^	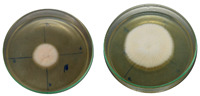		0.84
	Chloroquine 50 mg·L^−1^	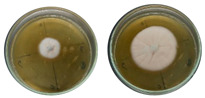		0.733
	Chloroquine 100 mg·L^−1^	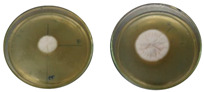		0.435
*Talaromyces verruculosus* CBMAI 2754	Control	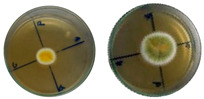		0.75
	Chloroquine 25 mg·L^−1^	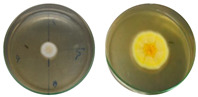		0.87
	Chloroquine 50 mg·L^−1^	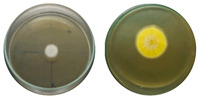		0.83
	Chloroquine 100 mg·L^−1^	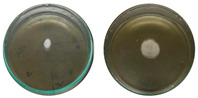		0.65
*Penicillium* cf. *guaibinense* CBMAI 2758	Control	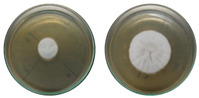		0.64
	Chloroquine 25 mg·L^−1^	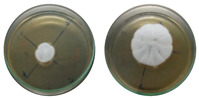		0.68
	Chloroquine 50 mg·L^−1^	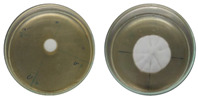		0.58
	Chloroquine 100 mg·L^−1^	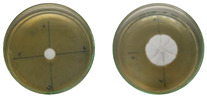		0.39

**Table 2:** The three independent variables used in the Box–Behnken factorial design.

Factors	Variables	Levels		
		−1	0	+1
X1	Time (days)	5	10	15
X2	pH	5	7	9
X3	Chloroquine diphosphate concentration (mg·L^−1^)	50	75	100

**Table 3:** Experimental design matrix and biodegradation responses of *Penicillium* cf. *guaibinense* CBMAI 2758.

Run	Levels of Uncoded and Coded Variables						Response (%BD)
	X1		X2		X3		
T1	5	−1	5	−1	75	0	>99.0
T2	15	1	5	−1	75	0	98.2
T3	5	−1	9	1	75	0	88.2
T4	15	1	9	1	75	0	91.2
T5	5	−1	7	0	50	−1	97.0
T6	15	1	7	0	50	−1	89.0
T7	5	−1	7	0	100	1	>99.4
T8	15	1	7	0	100	1	98.6
T9	10	0	5	−1	50	−1	95.5
T10	10	0	9	1	50	−1	87.9
T11	10	0	5	−1	100	1	93.9
T12	10	0	9	1	100	1	93.8
T13	10	0	7	0	75	0	92.8
T14	10	0	7	0	75	0	92.5
T15	10	0	7	0	75	0	91.7

**Table 4:** Computational toxicity parameters in animal models, with predictions of toxicity risks and tolerated doses of the potential carcinogen TopHit8.

	Computational Toxicological Parameters						Prediction of Toxicity Risks					Tolerated Doses of Potential Carcinogen		
Molecules							*Pimephales promelas*	*Daphnia magna*	Rat			Rat	Mouse	Rat
	Female/ Male		Ames	Skin		Ocular			Oral	Chronic	Inhalation	(Body Weight/Day)		
	Mouse	Rat	Mutagenicity	Irritancy	Sensitization	Irritation	LC_50_ (g/L)	EC_50_ (mg/L)	LD_50_ (g/kg)	LOAEL (g/kg)	LC_50_ (mg/m^3^/h)	TD_50_ (mg/kg)		RMTD (g/kg)
Chloroquine	C−	C−	M+	−	−	++++	0.009	11.40	1.068	0.062	24,400.2	0.376	12.73	0.016
M1	C−	C−	M−	−	−	+++	0.001	0.22	1.465	0.092	1511.87	3.394	28.74	0.093
M2	C	C−	M−	++	−	+++	2.56 × 10^−5^	0.32	0.853	0.007	2230.09	94.806	16.00	0.103
M3	C−	C−	M−	++	−	−	0.001	0.72	0.138	0.065	8395.07	3.020	27.90	0.392
M4	C−	C−	M−	−	+	+++	0.001	0.33	6.877	0.070	771.57	0.798	45.80	0.736

C− (non-carcinogen); M− (non-mutagen); M+ (mutagen); + (weak); ++ (mild); +++ (moderate); ++++ (severe).

**Table 5:** Binding affinity values of enzymes expressed by fungi with chloroquine.

Ligand	Binding Affinity (kcal/mol)		
	Phytase	Protease	Penicillopepsin-1
HIS	−7.208	--	--
PMS	--	−7.475	--
PP7	--	--	−9.547
Chloroquine	−7.097	−7.262	−7.923

## Data Availability

The data are contained in the article or Supplementary Material.

## Supplementary Materials

The following supporting information can be downloaded at https://www.mdpi.com/article/10.3390/jof11080579/s1: Figure S1: Morphology and molecular identification of filamentous fungi isolated from iron mine soil; Figure S2: RMSD representation of the crystallographic ligand (green) and best docking pose (gray) in the (A) Phytase; (B) Protease and (C) Penicillopepsin-1; Figure S3: Chromatographic analysis coupled to mass spectrometry (GC/MS) of the biodegradation reaction of chloroquine by the fungus *Penicillium* cf. *guaibinense:* CBMAI 2758; Table S1: Growth rates of the strains *Trichoderma pseudoasperelloides*, *T. verruculosus*, *Penicillium* cf. *guaibinense* and *Penicillium rolfsii* in the presence of chloroquine diphosphate, on 2% Sabouraud dextrose agar, 2% malt, pH 5, at 28 °C for 7 days; Table S2: Main ions observed in the mass spectra of metabolites identified from the biodegradation of chloroquine diphosphate (DCQ) by *Penicillium* cf. *guaibinense* CBMAI 2758.

## Abbreviations

The following abbreviations are used in this manuscript:

CBMAI	Brazilian Collection of Environmental and Industrial Microorganisms
pH	hydrogen of potential
EOMs	Emerging organic contaminants
GC-MS	Gas chromatography–mass spectrometry
SARS-CoV-2	Severe acute respiratory syndrome coronavirus 2
COVID-19	Coronavirus disease 2019
RGR	Radial growth rate
EPS	Extracellular polymeric substances
CTC	Chlortetracycline
NSAIDs	Non-steroidal anti-inflammatory drugs
CAS	Chemical Abstracts Service
HPLC	High-performance liquid chromatography
CPQBA	Multidisciplinary Center for Chemical, Biological, and Agricultural Research
DNA	Deoxyribonucleic acid
PCR	Polymerase chain reaction
HCl	Hydrochloric acid
KOH	Sodium hydroxide
DMSO	Dimethyl sulfoxide
BD	Biodegradation rate
RDC	Collegiate Board Resolution
ANVISA	National Health Surveillance Agency
RSD	Relative standard deviation
CV	Coefficient of variation
SD	Standard deviation
DMC	Determined mean concentration
LD	Limit of detection
LQ	Limit of quantification
