# Supplementary material for: Biodegradation of Chloroquine by a Fungus from Amazonian Soil, Penicillium guaibinense CBMAI 2758
Source: J Fungi (Basel). 2025 Aug 4;11(8):579. doi: 10.3390/jof11080579 (PMC12387243; doi:10.3390/jof11080579)
Supplement: Supplementary file 1 [file jof-11-00579-s001.zip › jof-3717448-supplementary.pdf]

Biodegradation of Chloroquine by a fungus from Amazonian Soil, *Penicillium guaibinense* CBMAI 2758

Patrícia de A. Nóbrega [1], Samuel Q. Lopes [1], Lucas S. Sá [1], Ryan da Silva Ramos [2], André Luiz M. Porto [3], Inana F. de Araújo [1], Fabrício H. e Holanda [4], Willian G. Birolli [5] and Irlon M. Ferreira [1,*]

1 Biocatalysis and Applied Organic Synthesis Laboratory, Federal University of Amapá, 68903-419 Macapá, AP, Brazil; doutoradobiotecnologia21@gmail.com (P.A.N); lucassa720@gmail.com (L.S.S.); inanafauro24@gmail.com (I.F.A.); irlon.ferreira@gmail.com (I.M.F.).

2 Post-Graduate Program in Biotechnology and Biodiversity - BIONORTE Network, Federal University of Amapá, Macapá, Amapá, Brazil.

3 Federal Institute of Science and Technology Education of Amapá, Rua Nilo Perçanha, Bairro Cajari, 68920-000 Laranjal do Jari, AP, Brazil.

4 Laboratory of Organic Chemistry and Biocatalysis, Institute of Chemistry of São Carlos, University of São Paulo, Ed. Prof. Douglas Wagner Franco, Av. João Dagnone 1100, Santa Angelina, 13563-120 São Carlos, SP, Brazil; almporto@iqsc.usp.br (A.L.M.P.)

5 Molecular Oncology Research Center, Institute of Learning and Research, Barretos Cancer Hospital, Av. Ébano 165-1, Dr. Paulo Prata, 14784-384 Barretos, SP, Brazil; willian_gb8@hotmail.com (W.G.B.)

* Correspondence: irlon.ferreira@gmail.com

Figure S1. Morphology and molecular identification of filamentous fungi isolated from iron mine soil: (A) *Trichoderma pseudoasperelloides* CBMAI 2752; (B) *Talaromyces verruculosus* CBMAI 2754 (C) *Penicillium rolfsii* CBMAI 2753 (D) *Penicillium* sp. cf. *guaibinense* CBMAI 2758. of four microorganisms based on *tef1* primers.

(A)

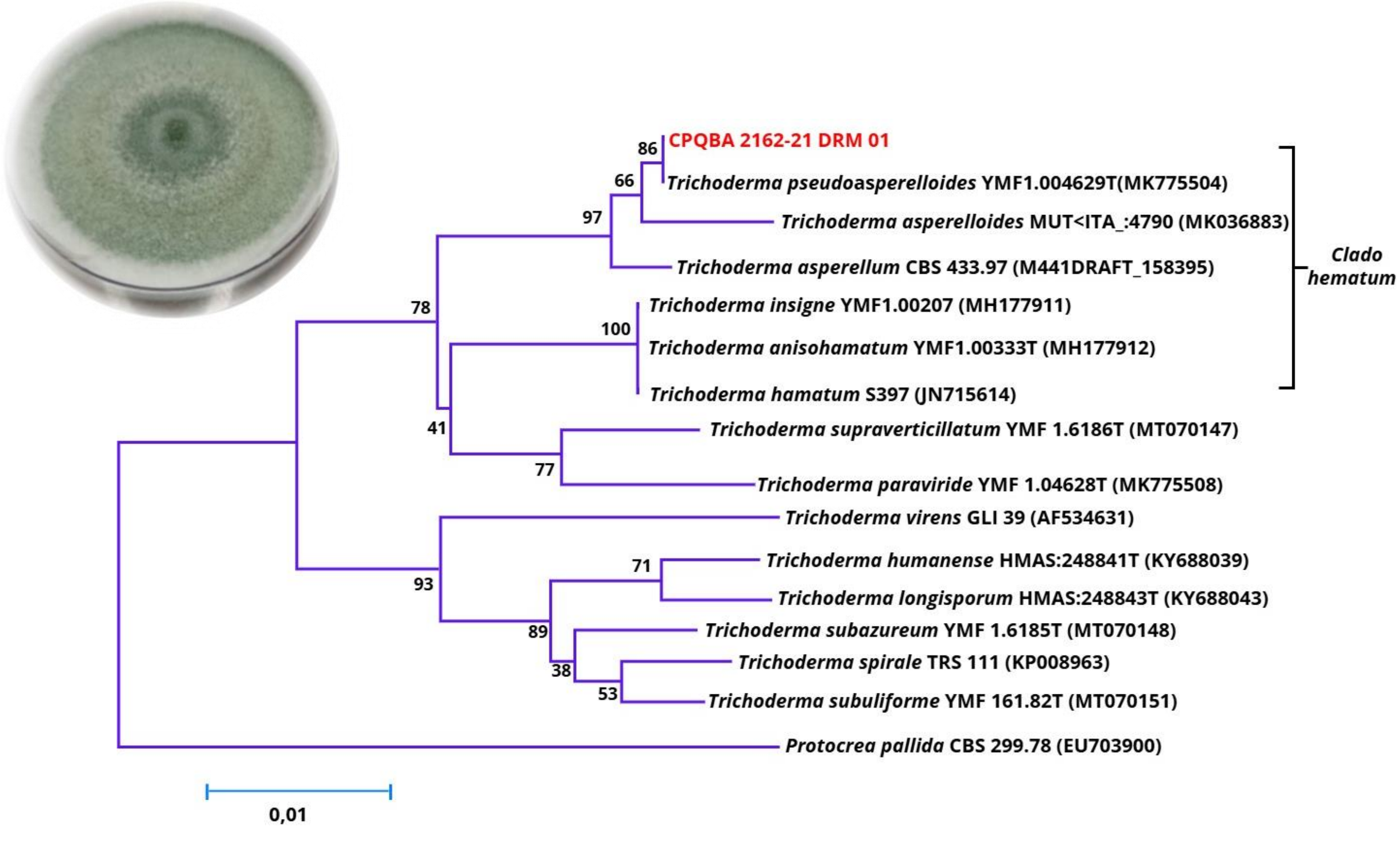

(B)

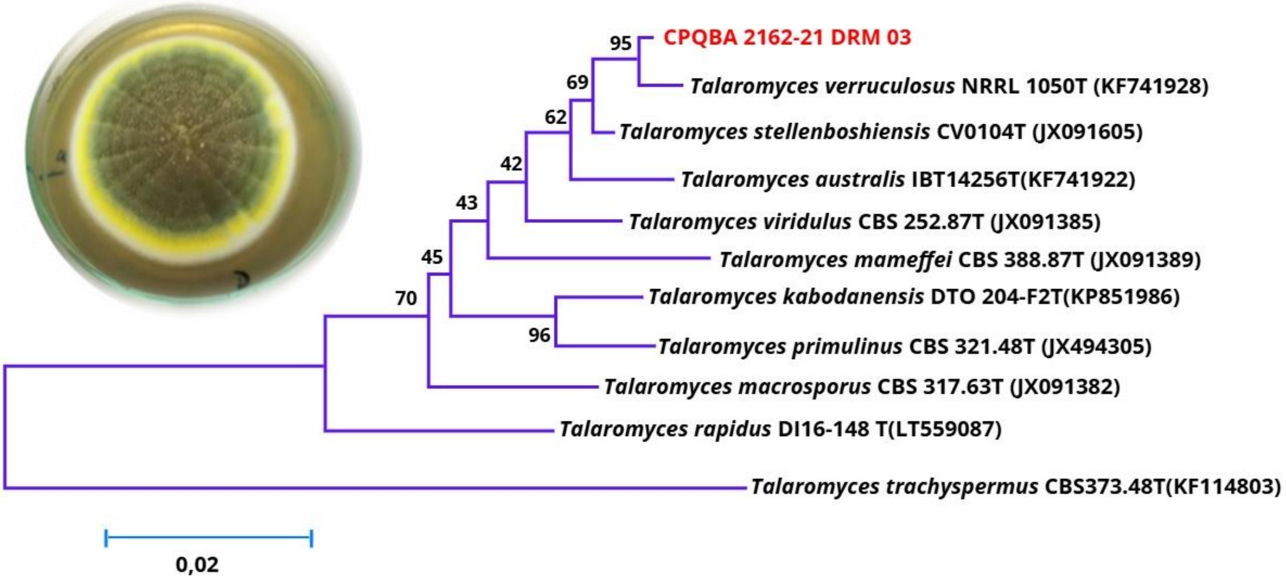
CPQBA 2162-21 DRM 03
Talaromyces verruculosus NRRL 1050T (KF741928)
Talaromyces stellenboshiensis CV0104T (JX091605)
Talaromyces australis IBT14256T(KF741922)
Talaromyces viridulus CBS 252.87T (JX091385)
Talaromyces mameffei CBS 388.87T (JX091389)
Talaromyces kabodanensis DTO 204-F2T(KP851986)
Talaromyces primulinus CBS 321.48T (JX494305)
Talaromyces macrosporus CBS 317.63T (JX091382)
Talaromyces rapidus DI16-148 T(LT559087)
Talaromyces trachyspermus CBS373.48T(KF114803)
95
69
62
42
43
45
70
96
0,02

(C)

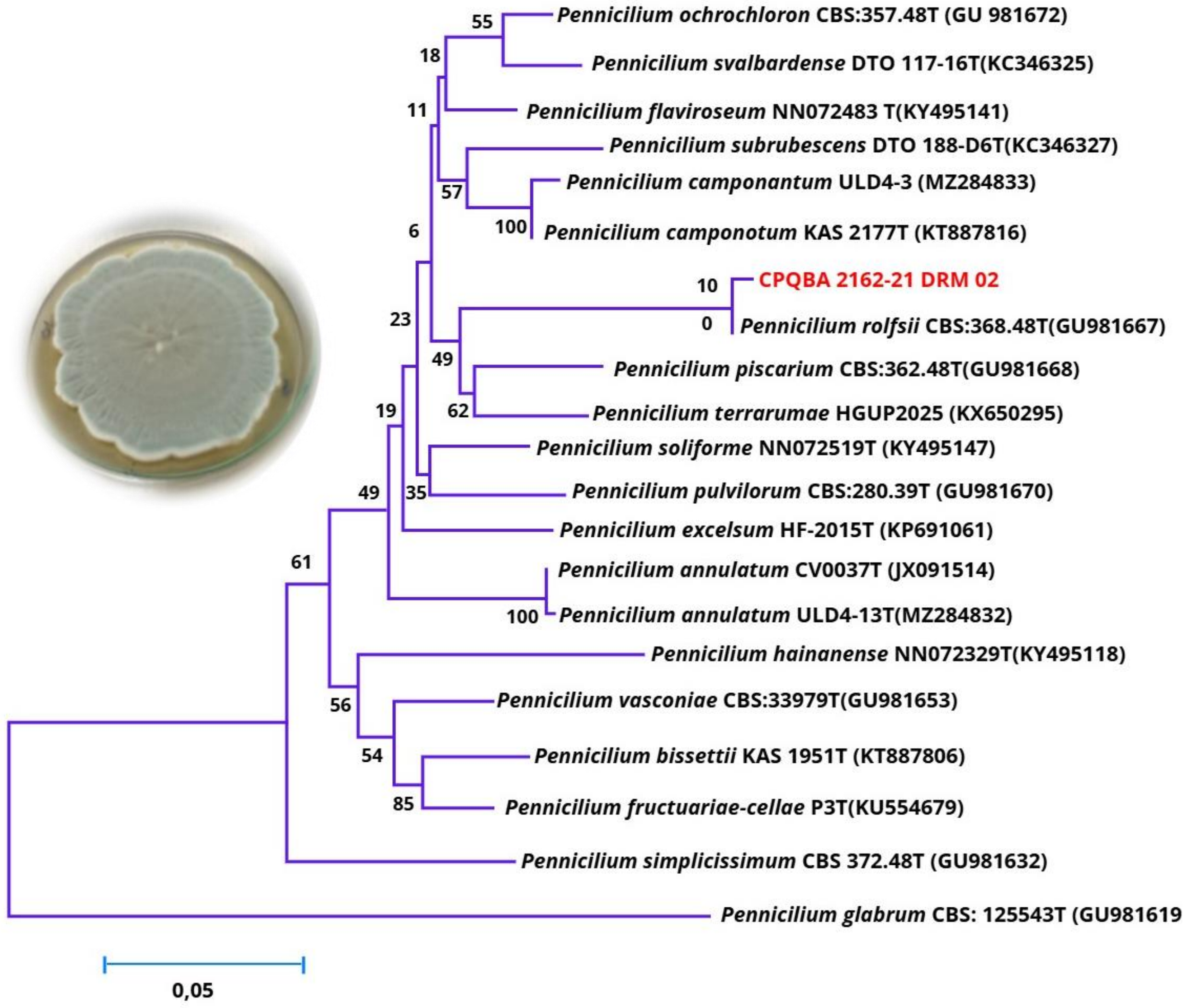
Pennicilium ochrochloron CBS:357.48T (GU 981672)
Pennicilium svalbardense DTO 117-16T(KC346325)
Pennicilium flaviroseum NN072483 T(KY495141)
Pennicilium subrubescens DTO 188-D6T(KC346327)
Pennicilium camponantum ULD4-3 (MZ284833)
Pennicilium camponotum KAS 2177T (KT887816)
CPQBA 2162-21 DRM 02
Pennicilium rolfsii CBS:368.48T(GU981667)
Pennicilium piscarium CBS:362.48T(GU981668)
Pennicilium terrarumae HGUP2025 (KX650295)
Pennicilium soliforme NN072519T (KY495147)
Pennicilium pulvilorum CBS:280.39T (GU981670)
Pennicilium excelsum HF-2015T (KP691061)
Pennicilium annulatum CV0037T (JX091514)
Pennicilium annulatum ULD4-13T(MZ284832)
Pennicilium hainanense NN072329T(KY495118)
Pennicilium vasconiae CBS:33979T(GU981653)
Pennicilium bissettii KAS 1951T (KT887806)
Pennicilium fructuariae-cellae P3T(KU554679)
Pennicilium simplicissimum CBS 372.48T (GU981632)
Pennicilium glabrum CBS: 125543T (GU981619
55
18
11
57
100
6
10
0
23
49
62
19
35
49
100
61
56
54
85
0,05

(D)

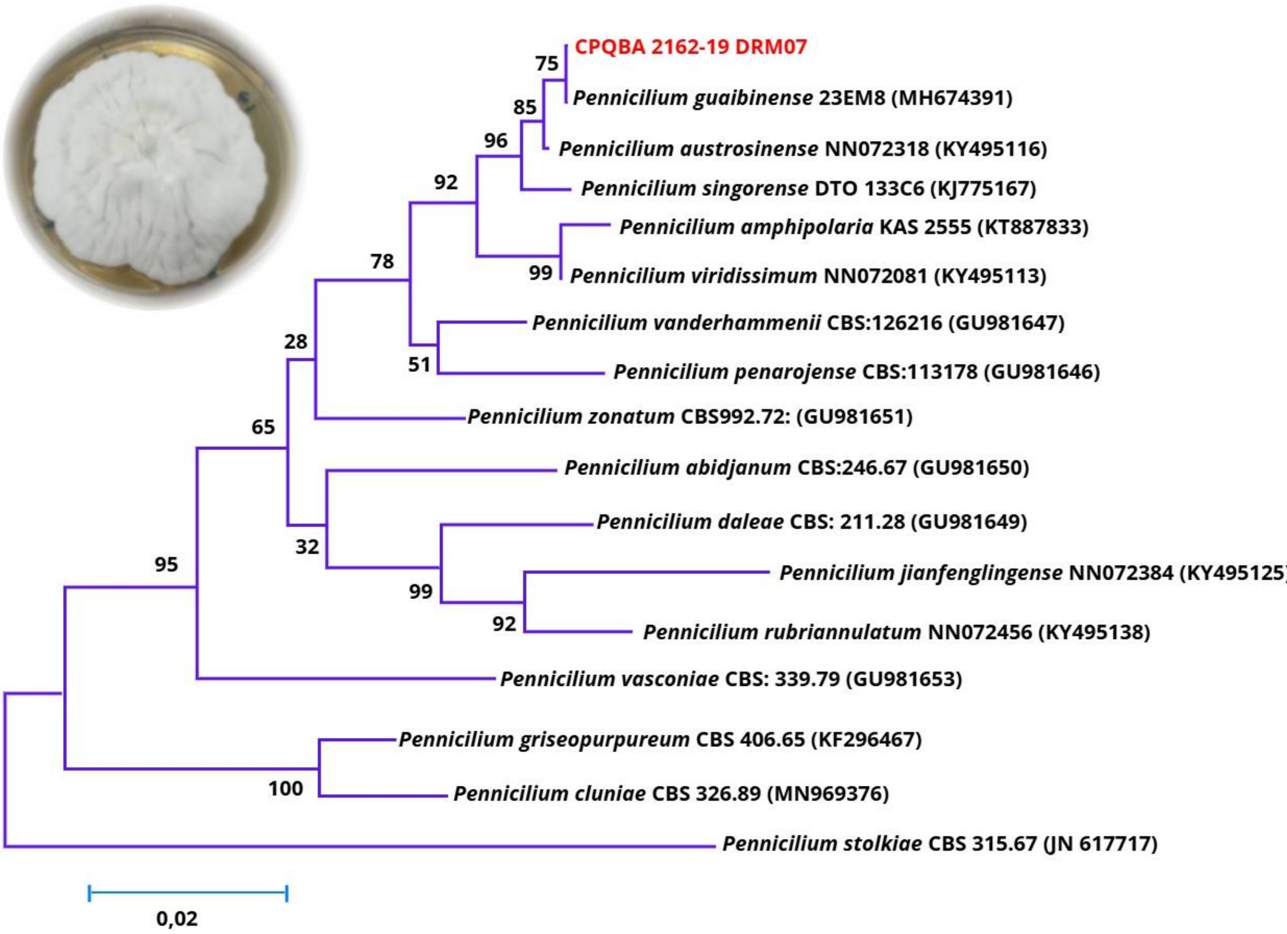

CPQBA 2162-19 DRM07
Pennicilium guaibinense 23EM8 (MH674391)
Pennicilium austrosinense NN072318 (KY495116)
Pennicilium singorense DTO 133C6 (KJ775167)
Pennicilium amphipolaria KAS 2555 (KT887833)
Pennicilium viridissimum NN072081 (KY495113)
Pennicilium vanderhammenii CBS:126216 (GU981647)
Pennicilium penarojense CBS:113178 (GU981646)
Pennicilium zonatum CBS992.72: (GU981651)
Pennicilium abidjanum CBS:246.67 (GU981650)
Pennicilium daleae CBS: 211.28 (GU981649)
Pennicilium jianfenglingense NN072384 (KY495125
Pennicilium rubriannulatum NN072456 (KY495138)
Pennicilium vasconiae CBS: 339.79 (GU981653)
Pennicilium griseopurpureum CBS 406.65 (KF296467)
Pennicilium cluniae CBS 326.89 (MN969376)
Pennicilium stolkiae CBS 315.67 (JN 617717)
75
85
96
92
78
99
51
28
65
32
99
92
95
100
0,02

Figure S2. RMSD representation of the crystallographic ligand (green) and best docking pose (gray) in the (A) Phytase; (B) Protease and (C) Penicillopepsin-1.

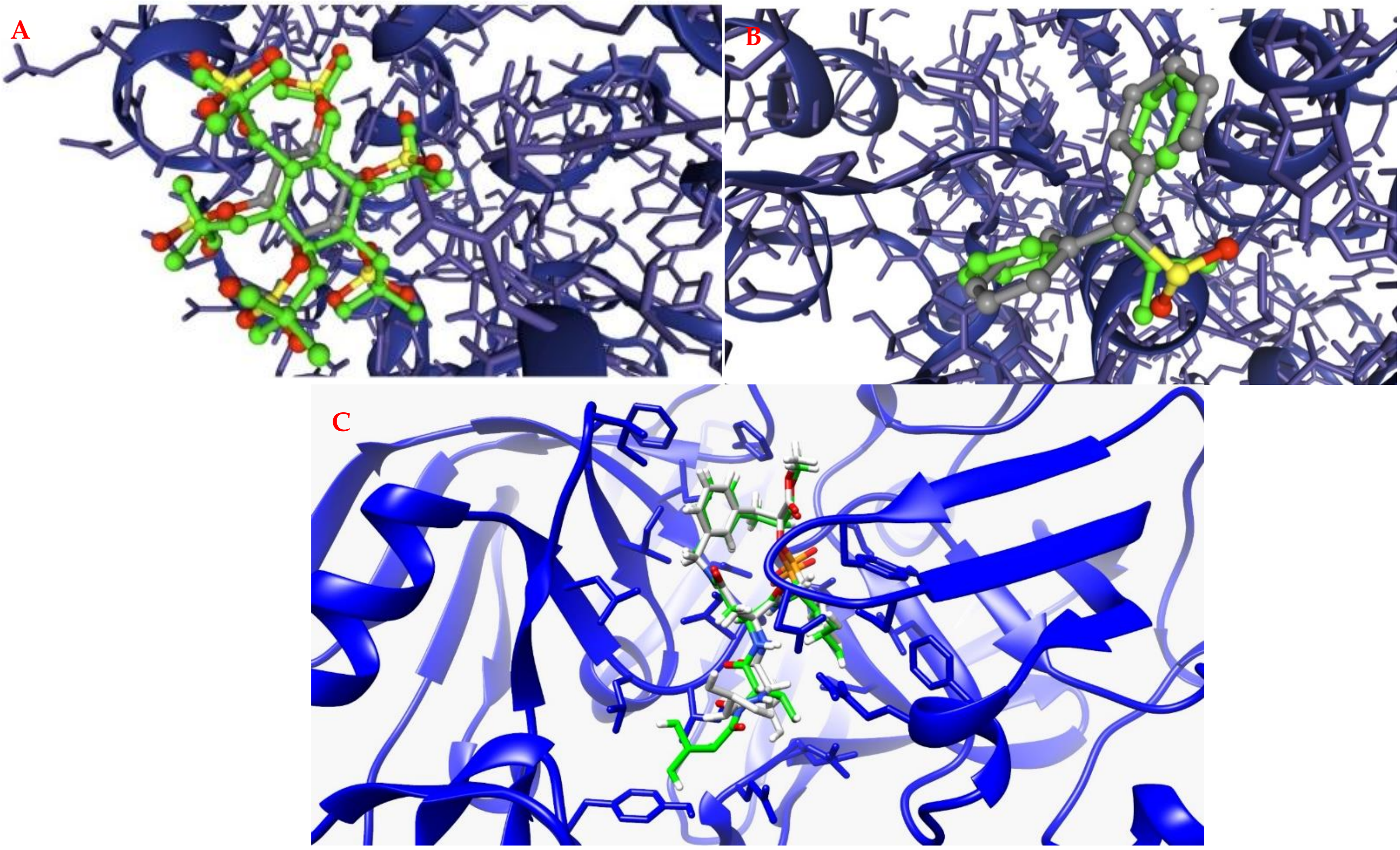

Table S1. Growth rates of the strains *Trichoderma pseudoasperelloides*, *T. verruculosus*, *Penicillium* cf. *guaibinense* and *Penicillium rolfsii* in the presence of chloroquine diphosphate, on 2% Sabouraud dextrose agar, 2% malt, pH 5, at 28 °C for 7 days.

Chloroquine (mg.L^{-1})	Equation	R^2	Growth speed (cm/d)
Trichoderma pseudoasperelloides CBMAI 2752			
25	Y=2.22x+0.229	0.992	2.22
50	Y=1.907x-0.266	0.992	1.9
100	Y=0.445x+0.271	0.992	0.44
Control	Y=3.100x-0.267	1	3.10
Talaromyces verruculosus CBMAI 2754			
25	Y=0.842x-0.452	0.998	0.84
50	Y=0.733x-0.529	0.998	0.733
100	Y=0.4355x-0.290	0.998	0.435
Control	Y=0.827x-0.126	0.997	0.82
Penicillium sp cf. *guaibinense* CBMAI 2758			
25	Y=0.876x-0.191)	0.99	0.87
50	Y=0.837x-0.438)	0.99	0.83
100	Y=0.651x-0.510)	0.99	0.65
Control	Y=0.755x+0.200	0.98	0.75
Penicillium rolfsii CBMAI 2753			
25	Y=0.682x+0.276	0.965	0.68
50	Y=0.581x+0.076	0.968	0.58
100	Y=0.393x-0.95)	0.999	0.39
Control	Y=0.640x+0.334	0.956	0.64

Figure S3. Chromatographic analysis coupled to mass spectrometry (GC/MS) of the biodegradation reaction of chloroquine by the fungus *Penicillium* sp. cf. *Guaibinense:* CBMAI 2758. *(a)* Chloroquine standard with the addition of the internal standard (a-naphthol). (*b*) Profile of metabolites identified during the biodegradation process.

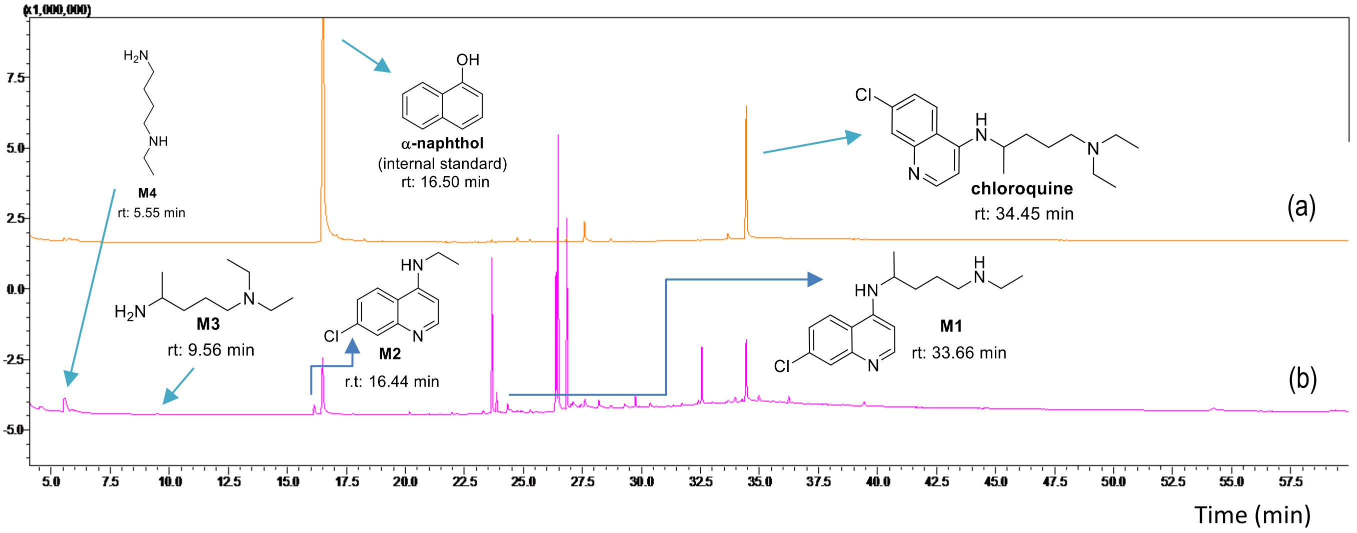

Table S2. Main ions observed in the mass spectra of metabolites identified from the biodegradation of chloroquine diphosphate (DCQ) by *Penicillium* sp. cf. *guaibinense* CBMAI 2758.

Metabolites	Spectrum from GC/MS analysis
**M1**	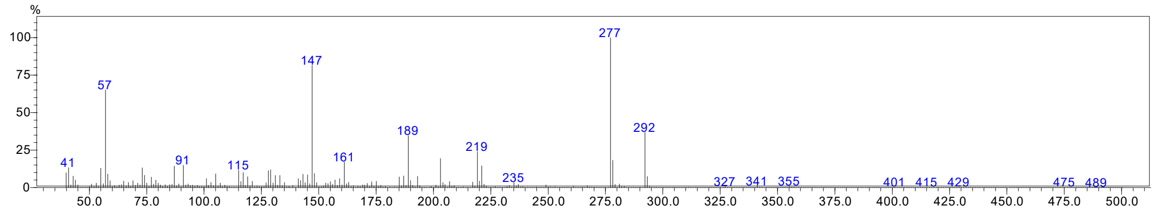
**M2**	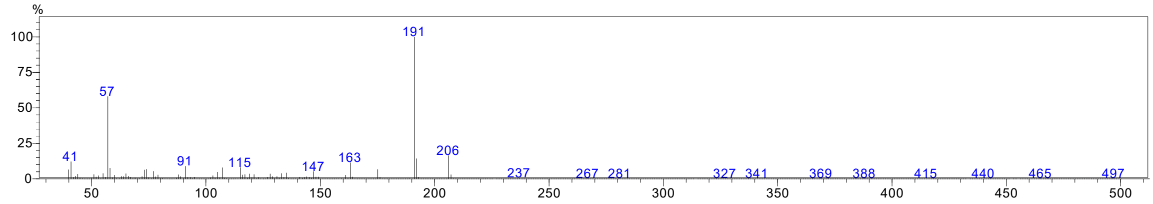
**M3**	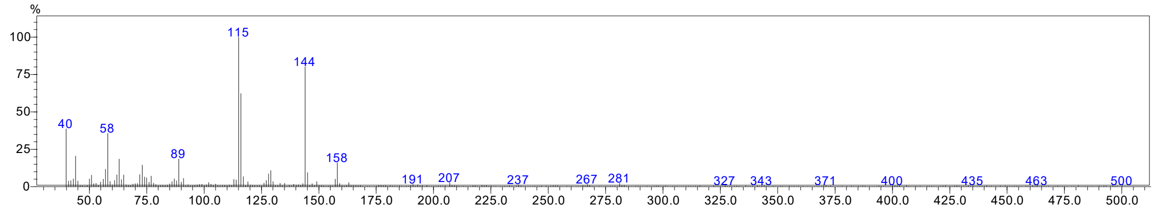
**M4**	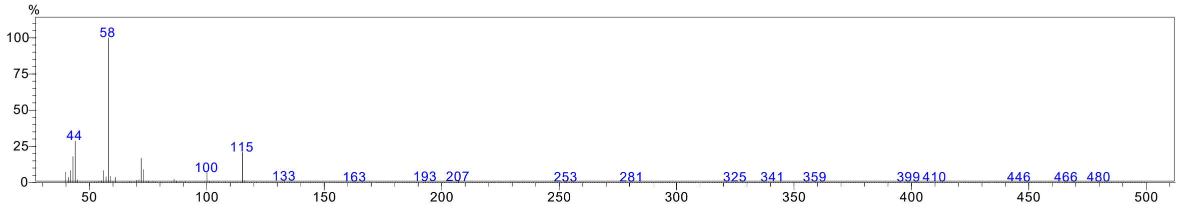
